# Retraction of: Sequence variations of 58 STRs and 94 SNPs in Northeastern Xibe with ForenSeq^TM^ DNA signature prep kit

**DOI:** 10.1093/fsr/owae030

**Published:** 2024-05-15

This is a retraction of: Fei Guo, Longnian Zhang, Yang Xin, Shiquan Liu, Sequence variations of 58 STRs and 94 SNPs in Northeastern Xibe with ForenSeq^TM^ DNA signature prep kit, *Forensic Sciences Research*, 2023, owad043, https://doi.org/10.1093/fsr/owad043

In December 2023, *Forensic Sciences Research* published the Accepted Manuscript of the article “Sequence variations of 58 STRs and 94 SNPs in Northeastern Xibe with ForenSeq^TM^ DNA Signature Prep Kit” by Fei Guo, Longnian Zhang, Yang Xin, and Shiquan Liu: https://doi.org/10.1093/fsr/owad043

In January 2024, the publisher was alerted to concerns regarding the applicability of the ethical approval for the research, the validity of the informed consent to participate in the research published in the article, and the validity of the informed consent to publish individual data presented in supplementary files to the article. The authors have not provided documentation demonstrating that they received sufficient ethical approval from a local ethical committee for the research, and as such the Editors-in-Chief and the Publisher have taken the decision to retract this paper. The authors have been informed of this decision and do not agree with the retraction. The supplementary files to the article have been removed given doubts over the validity of the informed consent to publish individual data.

